# Supplementary material for: A Critical View of the Application of the APEX Software (Aqueous Photochemistry of Environmentally-Occurring Xenobiotics) to Predict Photoreaction Kinetics in Surface Freshwaters
Source: Molecules. 2019 Dec 18;25(1):9. doi: 10.3390/molecules25010009 (PMC7017383; doi:10.3390/molecules25010009)
Supplement: Supplementary file 1 [file molecules-25-00009-s001.zip › 3.2.4_gcc-4.4.0.zip!/3.2.4_gcc-4.4.0/3.2.4_gcc-4.4.0/doc/octave/html/interpreter/Sparse-Matrices.html]

Sparse Matrices - Untitled


Next: Numerical Integration,
Previous: Diagonal and Permutation Matrices,
Up: Top


---

## 21 Sparse Matrices

- Basics: Creation and Manipulation of Sparse Matrices- Sparse Linear Algebra: Linear Algebra on Sparse Matrices- Iterative Techniques: Iterative Techniques- Real Life Example: Using Sparse Matrices
